# A comparison of robot-assisted and fluoroscopy-assisted kyphoplasty in the treatment of multi-segmental osteoporotic vertebral compression fractures

**DOI:** 10.7555/JBR.36.20220023

**Published:** 2022-05-10

**Authors:** Qingqing Li, Chaoqin Wu, Zhenfei Huang, Jiang Cao, Jie Chang, Guoyong Yin, Lipeng Yu, Xiaojian Cao, Tao Sui

**Affiliations:** Department of Orthopaedics, the First Affiliated Hospital of Nanjing Medical University, Nanjing, Jiangsu 210029, China

**Keywords:** spinal fracture, percutaneous kyphoplasty, robot-assisted

## Abstract

Osteoporotic vertebral compression fracture (OVCF) has become a major public health issue that becomes more pressing with increasing global aging. Percutaneous kyphoplasty (PKP) is an effective treatment for OVCF. Robot-assisted PKP has been utilized in recent years to improve accuracy and reduce complications. However, the effectiveness of robot-assisted PKP in the treatment of multi-segmental OVCF has yet to be proved. This study was designed to compare the efficacy of robot-assisted and conventional fluoroscopy-assisted multi-segmental PKP. A total of 30 cases with multi-segmental OVCF between April 2019 and April 2021 were included in this study. Fifteen cases were assigned to the robot-assisted PKP group (robot group) and 15 cases to the conventional fluoroscopy-assisted PKP group (conventional fluoroscopy group). The number of fluoroscopic exposures, fluoroscopic dose, operation time, cement leakage rate, visual analog scale (VAS) score, vertebral kyphosis angle (VKA), and height of fractured vertebral body (HFV) were compared between the 2 groups. The number of fluoroscopic exposures, fluoroscopic doses, and cement leakage rates in the robot group were lower than in the conventional fluoroscopy group (*P*<0.05) while the operative time in the robot group was longer than in the conventional fluoroscopy group (*P*<0.05). VAS score and VKA were decreased and HFV was increased after surgery in both groups (*P*<0.05). Therefore, robot-assisted PKP for the treatment of multi-segmental OVCF can reduce the number of fluoroscopic exposures, fluoroscopic doses, and cement leakage compared to conventional treatment. As such, robot-assisted PKP has good application prospects and is potentially more effective in the treatment of multi-segmental OVCF.

## Introduction

With an aging global population, osteoporotic vertebral compression fracture (OVCF) has become a major public health and social detriment, affecting the health and quality of life of the elderly^[1–3]^. It often leads to persistent pain and limited mobility, as well as a high rate of disability and death due to prolonged bed rest. Percutaneous kyphoplasty (PKP) has emerged as a minimally invasive surgical method for treating OVCF, achieving rapid stabilization of the vertebral body and good analgesic effects. By allowing patients to ambulate earlier, it greatly reduces the length of hospital stay as well as the bed-ridden complications, therefore greatly improving the patients' quality of life, and minimizing the medical and social burden^[4–5]^. However, the current PKP technique also brings about problems such as repeated fluoroscopic exposures, high fluoroscopic exposure dose and cement leakage^[6–8]^. To address these challenges, researchers have applied 3D printing technology, computer-aided navigation technology, digital design-assisted technology, and surgical robotics to improve the surgical efficacy of PKP^[9–18]^.

In recent years, the Tirobot robot has been used in PKP for OVCF in spinal surgery. Preliminary clinical results have shown that robotic assistance can reduce surgical trauma, fluoroscopic exposure, and cement leakage^[16–18]^. However, the efficacy of robot-assisted PKP for multi-segmental OVCF remains unclear. In this study, we compared the efficacy as well as the fluoroscopic exposure and radiological outcomes of Tirobot-assisted and conventional fluoroscopy-assisted PKP for multi-segmental OVCF.

## Patients and methods

### Case inclusion and exclusion criteria

The inclusion criteria were: 1) age ≥55 years; 2) bone mineral density T value ≤−2.5 standard deviations (SD); 3) imaging showing multi-segmental vertebral fractures without neurological symptoms, and with fracture segments consistent with the clinical examination; 4) diagnosis of OVCF, unresponsive to conservative treatment, with the patient's daily activities affected; and 5) compliance to surgical treatment with PKP as ordered. The exclusion criteria were: 1) vertebral tumor; 2) poor physical condition, or inability to tolerate surgery; and 3) healing from old vertebral fracture.

### Patient population

In this retrospective study, a total of 30 patients meeting the inclusion criteria were selected from a roster of OVCF cases treated between April 2019 and April 2021. Fifteen patients received Tirobot-assisted PKP (robot group) and 15 patients received conventional fluoroscopy-assisted PKP (conventional fluoroscopy group). All patients had minor or no obvious trauma. Preoperative X-rays, computed tomography (CT), magnetic resonance imaging, and bone density examinations were performed to confirm that the inclusion criteria were met. The procedure was approved by the ethical committee of the First Affiliated Hospital of Nanjing Medical University (Approval No. 2021-MD-100). Written informed consents were obtained from all the patients before conducting any procedures.

### Surgical techniques

Patients in both groups were placed in the prone position after general anesthesia.

**Figure 1:**
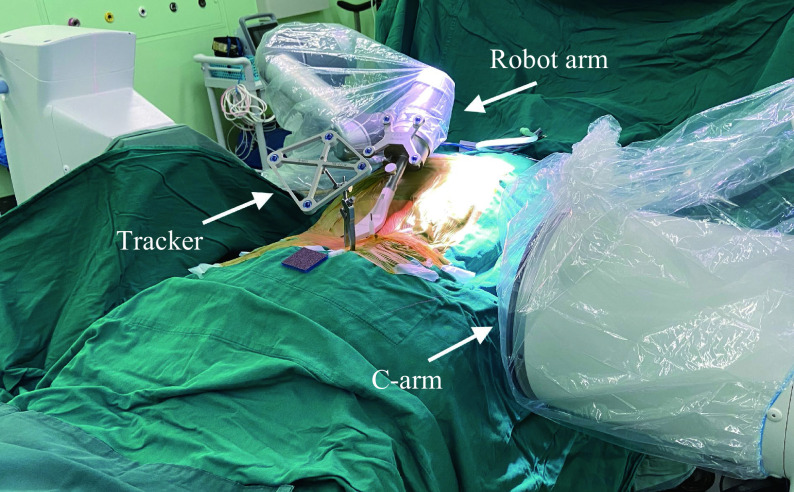
Schematic diagram of the use of TiRobot-assisted surgery.

Robot group: TiRobot system (Tinavi Medical Technologies Co., China) was used as previously detailed^[16]^. First, the tracer was fixed to the spinous process of the injured vertebrae in the caudal 2–3 segments with an original device and reinforced with a sterile film. A CT scan of the injured vertebra was performed, with the end of the robotic arm positioned above the injured vertebra and simultaneously in the center of the fluoroscopic field of the C-arm machine. A three-dimensional reconstruction was performed on the computer (***Fig. 1***). Next, preoperative planning was performed using the robotic system software to strategize the needle route into the vertebral body so that the tip of the puncture needle was in the anterior middle third of the vertebral body in the sagittal plane and in the center of the vertebral body in the transverse plane simultaneously. After planning the puncture route, the robot was programmed to remove the needle after delivering the pre-determined route, with a sharp knife breaking the skin and drilling the puncture needle through the sleeve. An expansion balloon was then placed and slowly expanded to bring the vertebral body to the desired height. The cement was prepared and pushed through the cement injector at the late stage of lasing, with slow, low-pressure, fractionated infusion, using the C-arm machine to fluoroscopically confirm the distribution of the cement on the lateral slice every 0.5 mL. The injection was stopped when the cement was distributed to the posterior 1/4 of the vertebral body. The working cannula and cement injector were rotated during the setting of the bone cement, and the working cannula and cement injector were withdrawn after the cement had set. The incision was subsequently disinfected, and the skin was sutured.

Conventional fluoroscopy group: Conventional C-arm was used to assist PKP according to the methods published in a previous study^[4]^. The C-arm was positioned in the frontal and lateral fluoroscopic view of the injured vertebra, and incisions were made at 3 to 4 centimeters on both sides of the injured vertebra with a length of 0.4 cm. Under fluoroscopy, the tip of the puncture needle was placed. When the lateral needle tip reached the posterior wall of the vertebral body and the orthotopic position was in the medial wall of the arch, the inner core was removed with the needle tip pointing below the anterior edge of the vertebral body. Subsequently, the guide needle was placed, and the working sleeve was replaced anterior to the posterior edge of the vertebral body. After the guide needle was removed, the working sleeve was retained. The drill was reamed to create an intraosseous tunnel, after which an expansion balloon was placed and slowly expanded to bring the vertebral body to the desired height. The process of cement injection was identical to that of the robot group.

### Outcome measures

The number of fluoroscopic exposures, fluoroscopic dose, operation time, cement injection volume, cement leakage rate, postoperative complications (neurological symptoms, infection, vascular embolism status), length of stay (LOS), visual analog scale (VAS) score, vertebral kyphosis angle (VKA) and height of fractured vertebral body (HFV) were recorded and compared between the two groups.

### Statistical analysis

SPSS 22.0 software was used for statistical analysis. Categorical data were presented as numbers and percentage values, with the chi-square test used to compare the differences between groups. The measurement data were expressed as mean±SD, and a Student's *t*-test or Mann-Whitney *U* test was used for comparing the differences between means. A repeated-measures ANOVA was used for comparison of measurement data at different time points within each group. Differences were considered statistically significant at *P*<0.05.

## Results

There was no significant difference identified in terms of age, gender, bone density value and number of fracture surgical segments between the conventional fluoroscopy group and the robot group (*P*>0.05,***Table 1***).

**Table 1:** Baseline of patients

Characters	Conventional fluoroscopy group (*n*=15)	Robot group (*n*=15)	*P*-value
Age (years)	69.3±8.4	69.7±8.1	0.895^#^
Gender Male/females (male/female)	3/12	2/13	0.624^*^
BMD (T score)	−3.4±1.5	−3.4±2.1	0.927^#^
Fracture segment distribution (T4/T5/T6/T7/T8/T9/T10/T11/T12/L1/L2/L3/L4/L5)	0/0/0/1/2/3/0/1/4/10/7/2/5/1	1/1/2/2/1/2/2/5/5/5/5/2/4/2	—
Number of fractured segments	2.4±0.5	2.6±0.6	0.35^#^
Data were presented as mean±SD. *P*-values were calculated by ^*^chi-square test or ^#^Student's *t*-test or Mann-Whitney *U* test. *P*<0.05 was considered statistically significant. BMD: bone mineral density.			

### Fluoroscopic exposure times and dose, operative duration, and postoperative complications

The number of fluoroscopic exposures, fluoroscopy dose and cement leakage rate in the robot group were lower than those in the conventional fluoroscopy group (*P*<0.05,***Table 2***). The operation time in the conventional fluoroscopy group was lower than that in the robot group (*P*<0.05,***Table 2***). There was no significant difference in the comparison of cement injection volume and LOS (*P*>0.05,***Table 2***) between groups. One patient in the robot group experienced intervertebral cement leakage, and nine patients in the conventional fluoroscopy group experienced intervertebral or paravertebral cement leakage. Neither of these patients experienced discomfort such as pain or neurological symptoms, thus no special treatments were performed. No postoperative complications (*e.g.*, infection or vascular embolism) occurred in either group.

**Table 2:** Fluoroscopic exposure times, fluoroscopic doses, operation time, cement injection volume, cement leakage rate, postoperative complications, and length of stay in the conventional fluoroscopic and robotic groups

Parameters	Conventional fluoroscopy group (*n*=15)	Robot group (*n*=15)	*P*-value
Fluoroscopic exposure times (*n*)	70.2±35.2	39.4±8.2	0.047^#^
Fluoroscopic doses (cGy.cm2)	435.4±119.4	222.2±95.1	0.006^#^
Operation time (minutes)	59.8±25.3	85.7±22.1	0.006^#^
Cement injection volume (mL)	5.2±1.3	4.9±1.3	0.544^#^
Cement leakage rate (%)	60.00	6.67	0.002^*^
Complications			
Neurological symptoms	0	0	-
Infection	0	0	-
Vascular Embolism	0	0	-
LOS (days)	1.7±1.0	2.2±1.4	0.290^#^
Data were presented as mean±SD or percentage values. *P*-values were calculated by ^*^chi-square test or ^#^Student's *t*-test or Mann-Whitney *U* test.*P*<0.05 was considered statistically significant. LOS: length of stay.			

### Preoperative and postoperative visual analog scores

The VAS scores in both groups at 1 day and 3 months postoperative follow-up were lower than corresponding pre-surgical values (*P*<0.05,***Table 3***). There was no significant difference in VAS scores 1 day and 3 months postoperatively (*P*>0.05,***Table 3***) for either group. There was no statistically significant difference in VAS scores between groups at any time points (*P*>0.05,***Table 3***).

**Table 3:** Preoperative and postoperative visual analog scores in the conventional fluoroscopic and robotic groups

Groups	Visual analog scores			*P*-value
	Preoperative	1 day postoperative	3 months postoperative	
Conventional fluoroscopy group (*n*=15)	7.1±0.7	2.6±0.5	2.4±0.6	<0.0001^*^
Robot group (*n*=15)	6.9±0.8	2.5±0.5	2.3±0.5	<0.0001^*^
*P*-value	0.363^#^	0.481^#^	0.749^#^	
Data were presented as mean±SD. *P*-values were calculated by ^*^one-way ANOVA for comparisons between different time points with a single variable or ^#^Student's *t*-test or Mann-Whitney *U* test. *P*<0.05 was considered statistically significant.				

### Preoperative and postoperative radiological findings

The VKA and HFV in both groups 1 d after surgery demonstrated significant improvement compared to post-operative values (*P*<0.05,***Table 4*** and ***Table 5***). There was no significant difference between the VKA in the robot group and in the conventional fluoroscopy group (*P*>0.05,***Table 4***). However, the HFV of the robot group was lower at preoperative baseline and 1 d after surgery than that of the conventional fluoroscopy group (*P*<0.05,***Table 5***). There was no difference in the postoperative HFV recovery between two groups (*P*>0.05,***Table 5***).

**Table 4:** Preoperative and postoperative vertebral kyphosis angle in the conventional fluoroscopic and robotic groups

Groups	Vertebral kyphosis angle (°)		*P*-value
	Preoperative	1 day postoperative	
Conventional fluoroscopy group (*n*=15)	8.6±4.9	5.3±4.2	<0.0001
Robot group (*n*=15)	8.6±4.7	4.7±3.9	<0.0001
*P*-value	0.977	0.589	
Data were presented as mean±SD. *P*-values were calculated by Student's *t*-test or Mann-Whitney *U* test. *P*<0.05 was considered statistically significant.			

**Table 5:** Preoperative and postoperative height of fractured vertebral body in the conventional fluoroscopic and robotic groups

Groups	HFV (cm)		Postoperative HFV recovery (cm)	*P*-value
	Preoperative	1 day postoperative		
Conventional fluoroscopy group (*n*=15)	18.9±3.6	21.8±3.6	2.9±2.0	<0.0001
Robot group (*n*=15)	12.8±4.7	15.5±4.6	2.7±1.7	<0.0001
*P*-value	<0.0001	<0.0001	0.750	
Data were presented as mean±SD. *P*-values were calculated by Student's *t*-test or Mann-Whitney *U* test. *P*<0.05 was considered statistically significant. HFV: height of fractured vertebral body.				

## Discussion

The occurrence of OVCF is a serious threat to the health and quality of life of the elderly^[1–3]^. PKP has evolved into a mature, effective, and safe minimally invasive surgical technique after decades of continuous improvement^[4–5]^. It has become the preferred treatment option for OVCF due to its rapid stabilization of the vertebral body, high pain relief rate, and facilitation of early ambulation after surgery. Currently, PKP relies on intraoperative C-arm fluoroscopy, requiring the surgeon to repeatedly adjust the puncture angle to ensure the safety of puncture, which not only increases the intraoperative fluoroscopic times and dose, but also increases the risk of cement leakage when the puncture angle deviates. Studies have shown that the increasing popularity of minimally invasive spine techniques has led to a significant increase in radiation exposure and radiation-induced complications for spinal surgeons, including cataracts, skin erythema, and malignancies, especially during the performance of multi-segmental minimally invasive procedures^[6–7]^. Therefore, experts have pointed out that spinal surgeons should minimize fluoroscopy to reduce radiation exposure and the associated complications. Many novel adjunctive techniques have been promoted to reduce intraoperative fluoroscopic exposure and increase puncture accuracy, such as digital subtraction angiography 3D reconstruction, computer-assisted navigation, spine surgery robots, digital design-assisted techniques, and 3D-printed transpedicular root puncture guide plates^[9–19]^. Among these, spinal surgery robots have been vigorously developed because they can implement real-time navigation to assist in surgery and overcome the data errors caused by changes in body position. Hence, the accuracy of robot-assisted surgery has improved more significantly than other assisted technologies. At present, mature robotic products that have been marketed worldwide include: the spinal surgical positioning system (Mazor Robotics Ltd., Israel) and the ROSA robot (Medtech S.A., France). In addition, the Tirobot developed by Tinavi Medical Technologies Company has already reached world-class level and is considered the only orthopaedic robot system that can perform limb, pelvic fracture, and full spinal surgery. Studies have shown that the nail placement accuracy of these three surgical robots is 98.3%, 97.3%, and 95.3% respectively, which is a significant improvement over the fluoroscopic-assisted nail placement accuracy of traditional C-arm machines^[20–22]^. Since PKP requires high accuracy of puncture depth and angle, these robots have been used to assist PKP for OVCF and have been linked to significant reductions of radiation exposure and cement leakage rate^[13–18]^. Despite this, the efficacy of the Tirobot-assisted PKP strategy in the treatment of multi-segmental OVCF remains unclear. In the present study, the VAS scores of patients with multi-segmental OVCF treated with either robot-assisted or conventional fluoroscopy-assisted surgical treatment demonstrated significantly improved post-operative scores at 1 day and 3 months follow-up. Patients in both groups had significantly better postoperative VKA and HFV measures than their pre-operative scores. While the HFV was lower in the robot group than in the conventional fluoroscopic group preoperatively and 1 day postoperatively, the 3 months post-operative HFV scores indicated that similar improvements could be achieved after intraoperative balloon expansion, significantly improving the posterior convexity angle of the vertebral body. These findings also suggest that in either group, balloons can be placed in an ideal position for expansion. The VAS and radiological results suggest that both groups achieved satisfactory surgical outcomes without neurological symptoms, infection, or vascular embolism, which is consistent with other reports^[13–18]^. Possible mechanisms for pain relief after PKP include restoration of vertebral body stability, axial stress bearing by the bone cement, reduction of fracture line micromotion, thermal and chemically toxic denervation of the bone cement, and correction of vertebral kyphosis by balloon expansion^[4,23]^.

We further compared intraoperative conditions and postoperative complications. The number of intraoperative fluoroscopies, total fluoroscopic radiation dose, and cement leakage rate were significantly lower in surgical cases performed with robot assistance than those performed with conventional fluoroscopic assistance. On the other hand, PKP with conventional fluoroscopic assistance observed shorter operative times than with the robotic-assisted method.

The non-visible nature of PKP has made the C-arm an indispensable tool for spine surgeons. In conventional PKP, the standard orthogonal position is based on the inferior border of the injured vertebral body, and a k-wire is used in the standard orthogonal position to puncture through the internal arch, during which multiple fluoroscopies are required to ensure that the puncture needle does not extend beyond the arch. For multi-segmental OVCF, this procedure needs to be repeated for each injured vertebra to ensure puncture accuracy. As a result, conventional fluoroscopy-assisted PKP for multi-segmental OVCF generates far more fluoroscopic exposure than single-segment OVCF, significantly increasing the risk of fluoroscopic radiation exposure to both surgeons and patients. We found that the Tirobot can plan the puncture paths of up to four consecutive vertebrae simultaneously after one 3D scan, which is expected to reduce this risk by avoiding repeated fluoroscopies to assess puncture entry points and angles.

In this study, it was demonstrated that Tirobot-assisted multi-segmental PKP surgery can significantly reduce the risk of radiation exposure by reducing their times and dose, especially for consecutive vertebral cases. After intra-operative 3D scanning and reconstruction, the robot system can plan multiple puncture paths for adjacent injured vertebrae simultaneously, eliminating the need for repeated fluoroscopies. The procedure also allows for the conversion of multi-segmental operations into a single segment. Thus, the treatment of consecutive multi-segmental OVCF would benefit more greatly from a robot-assisted design than of single-segmental OVCF.

To obtain a satisfactory puncture path, traditional PKP surgery often requires repeated fluoroscopies to adjust the puncture point and the head-tilt and inward-tilt angles of the puncture needle, which can easily damage the lateral wall of the pedicle or puncture the injured vertebral rupture, resulting in leakage of cement during pushing. This is significant as the current cement leakage rate during vertebroplasty has been reported between 23% to 62%^[15–16,24]^. Due to robot-assisted path planning, the puncture procedure of the robot group in this study was completed successfully in one session, reducing the cement leakage rate to 6.67%, significantly lower than the 60% rate observed in the conventional fluoroscopic group. These findings confirmed that precise puncture can significantly reduce the cement leakage rate. The operative time for robot-assisted surgical treatment of the spine varies widely depending on the segment and modality of the procedure and the stage of the learning curve at which the spine surgeon is placed^[25]^. In this study, the robot group required significantly more operative time than the conventional fluoroscopy group, likely due to the additional time required to set up and commission the robot, transfer the 3D scan data and reconstruct the model, and plan the optimal puncture path on the model. Moreover, when the injured spine segments were not adjacent to each other and were distributed in a salient pattern, a second 3D scan and puncture path planning were required, further increasing the operative time. We found that as the number of cases accumulated, the learning curve smoothed out and the operative time of the robot-assisted surgeries decreased slightly, though not enough to make a statistically significant difference.

Some of the limitations of this study included: 1. this study was a single-center, retrospective controlled study, and a multi-center prospective randomized controlled trial is needed to further validate the results; 2. the follow-up time of the patients was relatively short, and the observation of long-term efficacy and complications was insufficient; 3. the incidence of multi-segmental OVCF was low, subsequently the sample size was small, and the statistical results may have been biased; 4. this study only assessed visual analog scores. In the future, the Oswestry Disability Index, the Roland-Morris Disability Questionnaire for specific functional capacity, and the 36-Item Short-Form Health Survey for quality of life are needed to more comprehensively evaluate the clinical condition of patients.

In conclusion, the efficacy of Tirobot-assisted PKP for multi-segmental OVCF is stable and reliable. Despite an increase in operative time, the number of fluoroscopic exposures, fluoroscopic doses, and the incidence of cement leakage were significantly reduced. Robot-assisted PKP is safe and effective, having good application prospects. The procedure may be particularly advantageous for the treatment of continuous multi-segmental OVCF.
